# Heart Failure-Related Outcomes in Patients with Left Ventricular Dysfunction Undergoing Percutaneous Chronic Total Occlusion Revascularization

**DOI:** 10.31083/j.rcm2412345

**Published:** 2023-12-12

**Authors:** Pierluigi Lesizza, Lennert Minten, Ella Poels, Maarten Vanhaverbeke, Gianluca Castaldi, Keir McCutcheon, Daan Cottens, Carlo Zivelonghi, Pierfrancesco Agostoni, Christophe Dubois, Jo Dens, Johan Bennett

**Affiliations:** ^1^Department of Cardiovascular Medicine, University Hospitals Leuven, 3000 Leuven, Belgium; ^2^Department of Cardiovascular Sciences, Katholieke Universiteit Leuven, 3000 Leuven, Belgium; ^3^Department of Cardiology, Ziekenhuis Oost-Limburg, 3600 Genk, Belgium; ^4^Department of Cardiology, AZ Delta, 8800 Roeselare, Belgium; ^5^HartCentrum, Ziekenhuis Netwerk Antwerpen (ZNA) Middelheim, 2060 Antwerp, Belgium

**Keywords:** CTO PCI, LV dysfunction, heart failure

## Abstract

**Background:**

The presence of a chronic total occlusion (CTO) and severe left ventricular (LV) systolic dysfunction are known negative prognostic factors in patients with coronary artery disease. Several studies have examined the effect of CTO revascularization on mortality, symptoms, occurrence of myocardial infarction (MI), and cardiac function in patients with normal or reduced LV function. However, the effect of CTO revascularization on heart failure-related events in patients with LV dysfunction, such as heart failure hospitalization (HFH), the occurrence of atrial fibrillation (AF), and a worsening renal function (WRF), has not yet been evaluated. To assess the success rate and safety of CTO percutaneous coronary interventions (PCIs) in coronary patients with LV ejection fractions of ≤40% and evaluate the impact of successful CTO revascularization on HFH, occurrence of AF, and WRF.

**Methods:**

Prospectively, data were collected from CTO PCIs performed at three referral centers and analyzed. From a total of 1435 CTO PCIs, 132 (9.2%) patients with a left ventricular ejection fraction (LVEF) of ≤40% were included in this analysis. The median follow-up duration was 23.18 months (interquartile range (IQR): 11.02–46.66 months).

**Results:**

A successful CTO PCI was achieved in 109 of these patients, while the procedure was unsuccessful in 23 patients (82.5% procedural success rate). Overall, the intervention had an acceptable number of peri-procedural (or in-hospital) complications (9.1%). During the follow-up period, the rates of all-cause death, cardiovascular death, and non-fatal MI were not significantly different between the two groups. The rates of HFH were significantly lower in the successful PCI group, while WRF and AF did not differ between successful and unsuccessful PCI groups. Successful PCI and higher estimated glomerular filtration rate (eGFR) were independent predictors of a lower risk of HFH, while prior stroke and diabetes were independent predictors of a higher risk of HFH.

**Conclusions:**

In patients with reduced LV systolic function (ejection fraction, EF ≤40%), CTO PCI is a safe and effective procedure and successful CTO PCI is independently associated with a lower risk of HFH during follow-up. Further expansion of this cohort is necessary to confirm these results.

## 1. Introduction

Heart failure (HF) is a complex syndrome characterized by impaired cardiac function, leading to inadequate tissue perfusion and subsequent multiorgan dysfunction. It is a major cause of morbidity and mortality worldwide, with an increasing prevalence and economic burden. Left ventricular systolic dysfunction (LVSD), often results from coronary artery disease (CAD) and contributes significantly to the progression and clinical manifestations of HF, while the presence of LVSD is one of the strongest predictors of adverse clinical events among patients affected by CAD [1]. Despite advancements in pharmacological and device-based therapies, patients with LVSD continue to face substantial challenges and poorer long-term outcomes.

Coronary artery chronic total occlusions (CTOs), defined as complete occlusions of a coronary artery for at least three months, are a common finding in patients with CAD and LVSD, and their presence is associated with a worse prognosis [2, 3]. CTOs pose a unique therapeutic dilemma since traditional revascularization strategies, such as percutaneous coronary intervention (PCI), may be challenging owing to technical difficulties, a perceived lack of benefit [4, 5], and a possible higher incidence of stent failure, compared to non-CTO PCIs [6]. Consequently, patients with CTOs often receive medical management alone [7, 8, 9, 10], potentially limiting their prognosis and exacerbating HF symptoms.

Over the past decade, advances in interventional techniques, tools, and operator expertise have improved the success rates and safety profiles of CTO PCIs. Consequently, there has been a growing interest in exploring the potential benefits of CTO revascularization in patients with LVSD. Previous studies in this population [11, 12, 13] have focused on short-term procedural success, cardiovascular mortality, myocardial infarction, and angina relief; however, the long-term impacts of CTO PCIs on heart failure-related outcomes in this specific patient population remain largely unknown.

Understanding the effects of CTO PCI on heart failure-related outcomes in patients with LVSD is of paramount importance. Successful revascularization of CTOs has the potential to alleviate myocardial ischemia, improve left ventricular function, and subsequently, enhance HF symptoms and prognoses. However, the risks associated with CTO PCIs, including procedural complications, contrast-induced nephropathy, and potential exacerbation of HF, need to be carefully weighed against the expected benefits.

Therefore, the objective of this study was to examine the success rate and complication rate of PCIs for CTOs in patients with an LV ejection fraction ≤40% at baseline. We also aimed to explore the clinical outcomes during the follow-up of this cohort of patients, based on the success or failure of the CTO PCI, particularly, regarding heart failure-related clinical events, such as heart failure hospitalization (HFH), occurrence of atrial fibrillation (AF), and worsening of renal function (WRF).

## 2. Materials and Methods

### 2.1 Study Population

We conducted a prospective multicenter study that included all patients undergoing CTO PCIs at three Belgian hospitals from the beginning of each center’s CTO program (i.e., 2011, 2014, and 2017) until July 2022. Revascularization of CTOs was considered only for lesions supplying a viable myocardial territory and deemed clinically significant. The decision to perform the CTO PCI was taken by the local Heart team after a careful review of the clinical and imaging data for each patient.

### 2.2 Definitions and Objectives

Coronary CTOs were defined as angiographic evidence of total occlusion with a flow grade 0 Thrombolysis in Myocardial Infarction (TIMI) score in a major epicardial coronary artery of at least 2.5 mm, dating back at least 3 months [3]. The complexity of the CTO lesion and the difficulty of the attempt were evaluated using the J-CTO score [14]. Revascularization was considered complete if, before the CTO PCI procedure, all other hemodynamically significant vessels with a diameter >2.5 mm had been revascularized. Angiographic success was defined as final residual stenosis <30% (by visual estimation) and TIMI flow grade 3 after CTO recanalization. Procedural success was defined as angiographic success without major complications. Major complications were defined as in-hospital death, stroke, peri-procedural myocardial infarction (MI) requiring repeat catheterization, coronary perforation requiring treatment, major bleeding, or major vascular complications. Major vascular complications were defined as retroperitoneal hematoma, acute limb ischemia, and vascular bleeding, which required prolonged hospitalization or transfusion. Type 4 peri-procedural MI was defined as a high-sensitivity troponin T increase of at least 5 times the upper reference limit (URL) (13 ng/L), or a 20% increase and a URL >5 times if it was already elevated before PCI, in combination with ischemic symptoms or ischemic changes on the electrocardiogram (ECG). Contrast-induced nephropathy (CIN) was defined as either an absolute increase in serum creatinine concentration of 0.3 mg/dL compared to the reference concentration, a relative increase in serum creatinine concentration of 50% compared to the reference concentration, or a reduced urinary output of 0.5 mL/kg/hour for at least 6 hours, 24–72 hours after contrast administration. Cardiovascular deaths were defined as deaths caused by MI, sudden cardiac death, death caused by heart failure, deaths caused by stroke or cardiovascular hemorrhage, and deaths caused by other cardiovascular causes. HFH was defined as all hospitalizations caused by the occurrence of heart failure or worsening of pre-existing heart failure. AF was defined as any evidence of AF during the follow-up if AF was not present before or at the time of recruitment. WRF was defined as either the occurrence during follow-up of end-stage renal disease, a decrease of at least 50% in glomerular filtration rate (GFR), or a decrease of more than 30 mL/min/1.73 $m^{2}$ from the reference level to less than 60 mL/min/1.73 $m^{2}$ [15]. Follow-up data were censored 4 years after CTO PCI.

### 2.3 Interventional Procedure

Arterial access was generally established either via the right or left radial artery, or via the femoral artery if the radial approach was deemed unsuitable. The size of the guiding catheters used for the occluded artery was 7-F in most cases. Double injection was routinely considered in the presence of contralateral collaterals. The CTO crossing technique was divided into four categories: antegrade wire escalation (AWE), antegrade dissection and re-entry (ADR), retrograde wire escalation (RWE), and retrograde dissection and re-entry (RDR). AWE included true-to-true wiring, parallel wiring technique, and kissing wire technique. ADR involved subintimal tracking and distal re-entry (STAR), limited subintimal tracking (LAST), and the use of the CrossBoss and/or Stingray system (Boston Scientific, Marlborough, MA, USA). RDR included controlled antegrade and retrograde dissection (CART) and reverse CART. The initial CTO crossing technique and an eventual switch to another technique were decided based on the CTO hybrid algorithm and on the experience of the operator [16]. Antiplatelet therapy and heart failure medications were prescribed in accordance with the recommendations of the European Society of Cardiology [17].

### 2.4 Recruitment and Follow-up Assessment

Dyspnea and angina were assessed based on the New York Heart Association (NYHA) functional class and the Canadian Cardiovascular Society (CCS) class, respectively, before CTO PCI. LV function was evaluated by echocardiography. In the presence of normal wall motion or hypokinesia in the territory supplied by the CTO, no further viability testing was performed, while in patients with akinesia or dyskinesia in the CTO territory, viability assessment was performed by myocardial scintigraphy or cardiac magnetic resonance imaging. Each patient was evaluated at least once between 6 and 18 months after the intervention and clinical and echocardiographic characteristics were collected.

### 2.5 Statistical Analysis

Categorical data are presented with counts and frequencies (%) and compared by chi-square analysis; continuous data are described as mean ± standard deviation and analyzed by Student *t* test (2 groups). The time to event data four years after the CTO procedure were analyzed by Kaplan–Meir survival analysis and compared using the log-rank (Mantel–Cox) test, whenever appropriate. Missing data were very limited (<4%) and survival analysis considered the data of the right-censored subjects. To analyze the probability of heart failure hospitalization and cardiovascular death 4 years after the CTO procedure, two Cox proportional hazard models were built. The covariates in the model were identified using the best subset procedure among 18 clinically selected parameters, which were deemed important risk factors for worse outcomes following a CTO procedure. The model was built by backward selection based on the likelihood ratio method. All the statistical analyses were performed using GraphPad Prism version 9.5.1 (GraphPad Software, Inc., La Jolla, CA, USA) and SPSS version 28.0 (IMB, Armonk, NY, USA). *p* < 0.05 was considered statistically significant.

## 3. Results

### 3.1 Baseline Characteristics

Of the 1435 patients who underwent CTO PCI in the three centers, 132 (9.2%) had an LV ejection fraction ≤40%. Among these patients with reduced LV function, the mean age was 68.1 ± 11.5 years, and 107 patients (81.1%) were males. The mean ejection fraction was 30.9 ± 8.1%. The clinical, electrocardiographic, laboratory, and echocardiographic characteristics at recruitment are presented in Table 1.

**Table 1.** **Clinical, electrocardiographic, laboratory, and echocardiographic characteristics at baseline**.

		Entire cohort (n = 132)	Successful PCI (n = 109)	Unsuccessful PCI (n = 23)	Statistical difference (*p* value)
Age		68.10 ± 11.48	68.05 ± 11.44	68.35 ± 11.92	0.912
Male		107 (81.1)	88 (80.7)	19 (82.6)	0.835
BMI, ${\mathrm{kg/m}}^{2}$		27.49 ± 4.73	27.30 ± 4.90	28.40 ± 3.79	0.237
Diabetes		48 (36.4)	39 (35.8)	9 (39.1)	0.761
Current smoker		31 (23.5)	25 (22.9)	6 (26.1)	0.746
Hypertension		94 (71.2)	79 (72.5)	15 (65.2)	0.485
Dyslipidemia		116 (87.9)	93 (85.3)	23 (100)	0.050
Peripheral vascular disease		30 (22.7)	26 (23.9)	4 (17.4)	0.502
Chronic kidney disease		60 (45.5)	43 (39.4)	17 (73.9)	0.003
Prior MI		93 (70.5)	76 (69.7)	17 (73.9)	0.689
Prior PCI		66 (50)	52 (47.7)	14 (60.9)	0.251
Prior CABG		24 (18.2)	17 (15.6)	7 (30.4)	0.094
Prior stroke		7 (5.3)	6 (5.5)	1 (4.3)	0.822
Prior HFH		54 (41.2)	40 (37.0)	14 (60.9)	0.035
Number of vessels involved					0.182
	1	48 (36.4)	40 (36.7)	8 (34.8)	
	2	45 (34.1)	43 (39.4)	2 (8.7)	
	3	39 (29.5)	26 (23.9)	13 (56.5)	
NYHA class					0.382
	1	30 (24.6)	28 (27.2)	2 (10.5)	
	2	51 (41.8)	44 (42.7)	7 (36.8)	
	3	35 (28.7)	18 (27.2)	7 (36.8)	
	4	6 (5.9)	3 (2.9)	3 (15.8)	
CCS class					0.538
	No angina	71 (54.2)	56 (51.9)	15 (65.2)	
	1	25 (19.1)	22 (20.4)	3 (13.0)	
	2	22 (16.8)	20 (18.5)	2 (8.7)	
	3	13 (9.9)	10 (9.3)	3 (13.0)	
	4	0 (0.0)	0 (0.0)	0 (0.0)	
Systolic blood pressure, mmHg		126.85 ± 24.99	126.02 ± 23.29	130.14 ± 31.24	0.568
Heart rate, bpm		74.51 ± 16.73	74.07 ±15.71	76.64 ± 21.26	0.596
Sinus rhythm		108 (81.8)	90 (82.6)	18 (78.3)	0.626
Left bundle branch block		23 (17.7)	18 (16.8)	5 (21.7)	0.575
Creatinine, mg/dL		1.27 ± 0.79	1.26 ± 0.84	1.29 ± 0.46	0.828
eGFR, mL/min/1.73 $m^{2}$		70.03 ± 27.92	71.39 ± 22.83	63.22 ± 22.15	0.153
Hemoglobin, g/dL		13.43 ± 1.80	13.59 ± 1.72	12.69 ± 2.02	0.055
Sodium, mmol		139.32 ± 3.49	139.27 ± 3.59	139.55 ± 3.04	0.708
Left ventricular ejection fraction, %		31.41 ± 8.13	31.49 ± 8.13	31.05 ± 8.35	0.823
Right ventricular dysfunction		25 (22.3)	22 (23.9)	3 (15.0)	0.386
Mitral regurgitation grade >2		23 (19.3)	18 (18.6)	5 (22.7)	0.655
Tricuspid regurgitation grade >2		9 (8.0)	5 (5.4)	4 (20.0)	0.028
Treatment					
	ACEi/ARB/ARNI	103 (78.0)	84 (77.1)	19 (82.6)	0.559
	Beta-blocker	111 (84.7)	92 (85.2)	19 (82.6)	0.755
	ARA	63 (48.1)	50 (46.3)	13 (56.5)	0.373
	Diuretics	65 (49.6)	51 (47.2)	14 (60.9)	0.235
	Digoxin	6 (4.6)	6 (5.6)	0 (0.0)	
ICD		19 (14.4)	18 (16.5)	1 (4.3)	0.316
CRT		5 (3.8)	2 (1.8)	3 (13.0)	0.011

BMI, body mass index; MI, myocardial infarction; PCI, percutaneous coronary intervention; CABG, coronary artery bypass grafting; HFH, heart failure hospitalization; NYHA, New York Heart Association; CCS, Canadian Cardiovascular Society; eGFR, estimated glomerular filtration rate; ACEi, angiotensin-converting enzyme inhibitor; ARB, angiotensin receptor blocker; ARA, aldosterone receptor antagonist; ICD, implantable cardioverter-defibrillator; CRT, cardiac resynchronization therapy; ARNI, Angiotensin receptor/neprilysin inhibitor.

The characteristics were homogeneous in both groups, except for a statistically significant higher prevalence of chronic kidney disease (CKD), prior HFH and cardiac resynchronization therapy (CRT) implantations, and lower prevalence of complete functional revascularization before index procedure in the unsuccessful PCI group.

The baseline angiographic characteristics are presented in Table 2. The unsuccessful CTO PCIs, as expected, had more challenging lesions to treat and presented a significantly higher J-CTO score.

**Table 2.** **Angiographic characteristics**.

		Entire cohort	Successful PCI (n = 109)	Unsuccessful PCI (n = 23)	Statistical difference (*p* value)
Target vessel					
	LAD	47 (35.6)	39 (35.8)	8 (34.8)	0.928
	LCx	28 (21.2)	26 (23.9)	2 (8.7)	0.106
	RCA	61 (46.2)	49 (45.0)	12 (52.2)	0.528
	LM	3 (2.3)	2 (1.8)	1 (4.3)	0.462
Complete revascularization before CTO PCI		72 (54.5)	65 (59.6)	7 (30.4)	0.011
Blunt stump		64 (48.5)	46 (42.2)	18 (78.3)	0.002
Bending >45°		48 (36.4)	35 (32.1)	13 (56.5)	0.027
Severe calcifications		49 (52.1)	38 (47.5)	11 (78.6)	0.032
CTO length, mm		22.14 ± 12.65	21.29 ± 11.86	26.17 ± 15.53	0.083
CTO length ≥20 mm		82 (62.1)	64 (58.7)	18 (78.3)	0.079
In-stent CTO		11 (8.3)	8 (7.3)	3 (13.0)	0.368
Prior attempt		14 (10.6)	11 (10.1)	3 (13.0)	0.676
J-CTO score ≥3		11 (8.3)	8 (7.3)	3 (13.0)	0.368
J-CTO score		2.11 ± 1.25	1.92 ± 1.20	3.04 ± 1.07	<0.001

LAD, left anterior descending artery; LCx, left circumflex artery; RCA, right coronary artery; LM, left main; CTO, chronic total occlusion; PCI, percutaneous coronary intervention.

### 3.2 Procedural Characteristics and Complications

The procedural characteristics are presented in Table 3. Angiographic success and procedural success were achieved in 116 patients (87.9%) and 109 patients (82.5%), respectively. Unsuccessful CTO PCIs had a significantly longer fluoroscopy time compared to successful CTO PCIs; however, the skin dose, although higher, was not statistically different. Overall, the effective crossing techniques were as follows: AWE in 108 patients, ADR in 4 patients, RWE in 1 patient, and RDR in 4 patients. The incidence of any complication was acceptable (Table 4), with 12 (9.1%) patients experiencing complications, and there was 1 in-hospital death (not procedure-related).

**Table 3.** **Procedural characteristics**.

	Entire cohort	Successful PCI (n = 109)	Unsuccessful PCI (n = 23)	Statistical difference (*p* value)
Number of stents	2.06 ± 1.06	2.14 ± 1.04	1.20 ± 0.92	0.011
Stent length, mm	63.76 ± 33.09	65.09 ± 32.90	45.75 ± 32.31	0.141
Procedure time, min	97.00 ± 47.50	95.66 ± 48.09	103.59 ± 44.97	0.462
Fluoroscopy time, min	34.22 ± 21.17	32.23 ± 21.06	43.65 ± 19.47	0.017
Contrast volume, mL	245.50 ± 104.33	244.67 ± 107.92	249.30 ± 87.85	0.827
Skin dose, mGray	1751.35 ± 1806.95	1641.99 ± 1701.28	2260.13 ± 2207.03	0.216

PCI, percutaneous coronary intervention.

**Table 4.** **Peri-procedural complications**.

	Entire cohort	Successful PCI (n = 109)	Unsuccessful PCI (n = 23)	Statistical difference (*p* value)
Patients with at least one complication	12 (9.1)	4 (3.7)	8 (34.8)	<0.001
Coronary perforation	3 (2.3)	2 (1.8)	1 (4.3)	0.462
Pericardial effusion	3 (2.3)	1 (0.9)	2 (8.7)	0.023
In-hospital death	1 (0.8)	0 (0.0)	1 (4.3)	0.029
Stroke	0 (0.0)	0 (0.0)	0 (0.0)	/
Major bleeding	1 (0.8)	0 (0.0)	1 (4.3)	0.029
Retroperitoneal hematoma	0 (0.0)	0 (0.0)	0 (0.0)	/
Hematoma >5 cm	0 (0.0)	0 (0.0)	0 (0.0)	/
Acute limb ischemia	1 (0.8)	0 (0.0)	1 (4.3)	0.029
CIN	5 (3.8)	2 (1.8)	3 (13.0)	0.011
Peri-procedural myocardial infarction	7 (5.3)	3 (2.8)	4 (17.4)	0.004

CIN, contrast-induced nephropathy; PCI, percutaneous coronary intervention.

### 3.3 Clinical Status during Follow-up

Follow-up data were available for 131 patients (1 patient died during the same hospitalization). The median follow-up duration was 23.18 months (interquartile range (IQR): 11.02–46.66 months). The NYHA and CCS patient classes showed significant improvements compared to the baseline, although there was no difference between the successful and unsuccessful CTO PCIs (**Supplementary Table 1**). Left ventricular ejection fraction (EF) demonstrated improvement compared to baseline, although there was no significant difference between the successful and unsuccessful PCIs (at baseline 31.49 ± 8.13% and 31.05 ± 8.35% in the successful and not successful PCI group, respectively, at follow-up 40.1 ± 11.3% and 35.4 ± 8.6%, respectively). During the follow-up period, adherence to optimal medical treatment for heart failure remained high.

### 3.4 Clinical Events during Follow-up

**Fig. 1.**
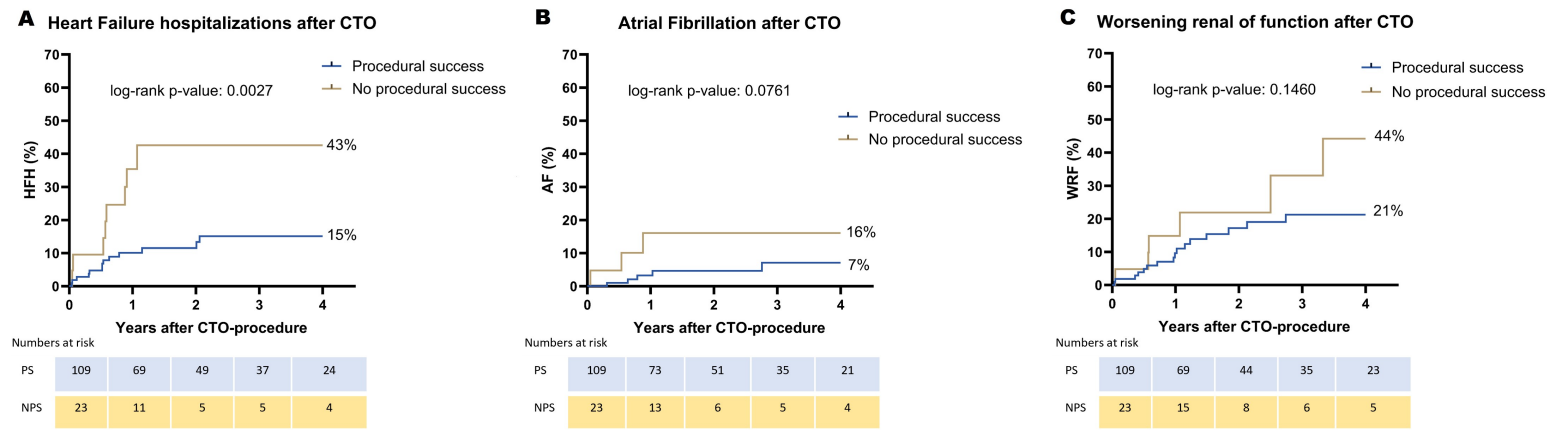
**Heart failure-related events during follow-up**. CTO, chronic total occlusion; AF, atrial fibrillation; HFH, heart failure hospitalization; WRF, worsening renal function.

The median follow-up duration was 23.18 months (interquartile range: 11.02–46.66 months). The events during the follow-ups are summarized in Table 5, Fig. 1, and **Supplementary Fig. 1**. There were no significant differences in the rates of all-cause death, cardiovascular death, non-cardiovascular death, and non-fatal myocardial infarction. Among these, there was a trend toward a lower rate of cardiovascular deaths in the successfully revascularized patients (at 4 years 14% vs. 23% in the successful and non-successful PCI groups, respectively, *p* = 0.0574). HFH was significantly lower in the successful PCI group (15% vs. 43%, *p* = 0.0027). There was a lower incidence of WRF and AF in the successful PCI group; however, it was not significantly different.

**Table 5.** **Clinical events during follow-up**.

	Entire cohort	Successful PCI (n = 108)	Unsuccessful PCI (n = 23)	Statistical difference (*p* value)
Death	25 (19.1)	20 (18.5)	5 (21.7)	0.721
Cardiovascular death	15 (11.5)	10 (9.3)	5 (21.7)	0.088
Non-cardiovascular death	10 (7.6)	10 (9.3)	0 (0.0)	0.129
Non-fatal myocardial infarction during follow-up	4 (3.1)	3 (2.8)	1 (4.3)	0.691
Target vessel revascularization	6 (4.6)	5 (4.6)	1 (4.3)	0.953
Stroke	3 (2.3)	1 (1.9)	1 (4.3)	0.467
HFH	21 (16.0)	13 (12.0)	8 (34.8)	0.007
WRF	23 (17.7)	17 (15.9)	6 (26.1)	0.245
Atrial fibrillation	8 (6.2)	5 (4.7)	3 (13.0)	0.130
Sudden cardiac death/ventricular fibrillation/sustained ventricular tachycardia/appropriate ICD intervention	11 (8.4)	8 (7.4)	3 (13.0)	0.376

Median follow-up: 23.18 months (interquartile range: 11.02–46.66 months). HFH, heart failure hospitalization; WRF, worsening renal function; ICD, implantable cardioverter-defibrillator; PCI, percutaneous coronary intervention.

**Fig. 2.**
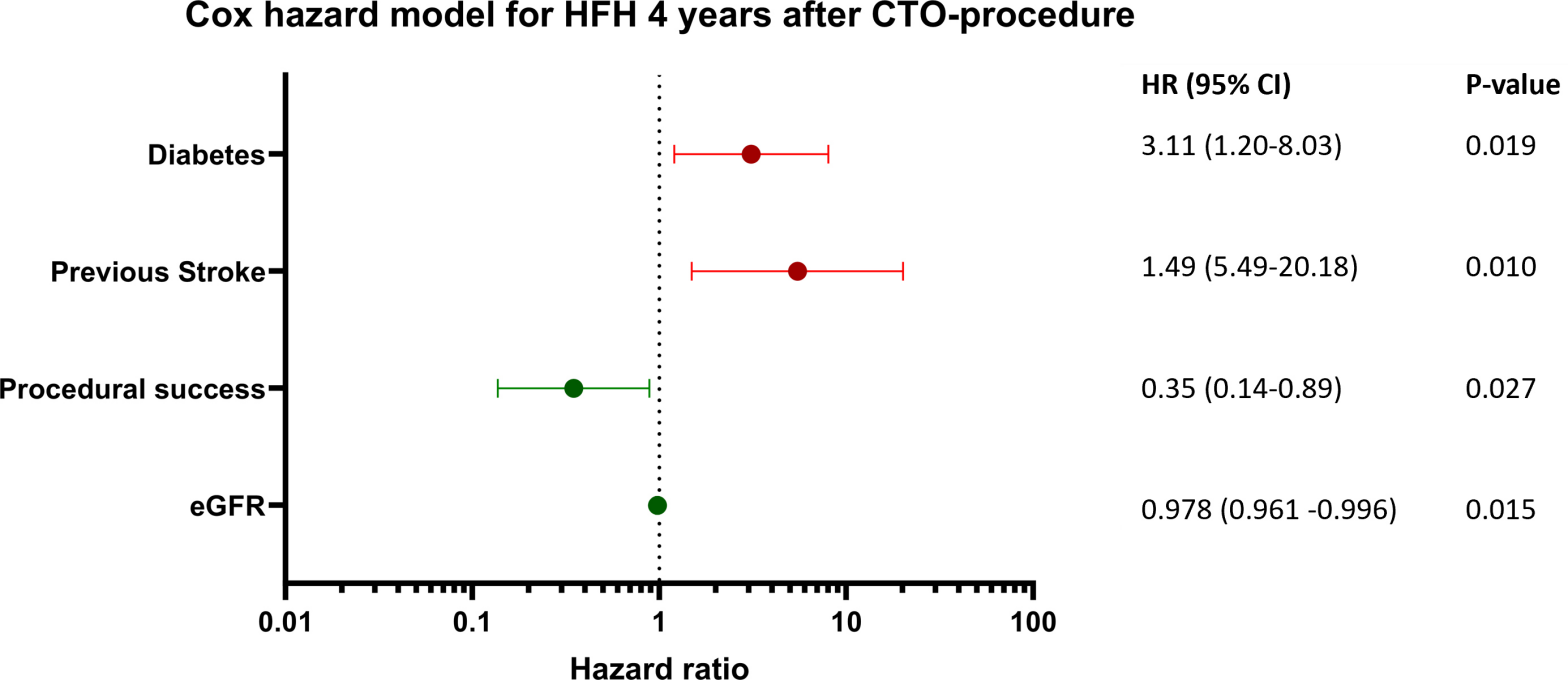
**Cox hazard model for heart failure admissions 4 years after CTO procedure**. HFH, heart failure hospitalization; CTO, chronic total occlusion; eGFR, estimated glomerular filtration rate; HR, hazard ratio.

Multivariate analysis showed that previous stroke and diabetes were independent predictors of HFH (HR 3.110, 95% CI 1.204–8.033, *p* = 0.019 for diabetes, HR 5.490, 95% CI 1.494–20.177, *p* = 0.010 for stroke), whereas higher eGFR and successful PCI were independently associated with a lower rate of HFH (HR 0.978, 95% CI 0.961–0.996, *p* = 0.015 for each point eGFR, HR 0.349, 95% CI 0.137–0.886, *p* = 0.027 for procedural success) (Table 6 and Fig. 2).

**Table 6.** **Cox hazard model for HFH 4 years after CTO PCI**.

Characteristics	Univariate hazard ratio	95% Confidence interval	*p*-value	Multivariate hazard ratio	95% Confidence interval	*p*-value
Age, years	1.006	0.969–1.045	0.751			
Male gender	1.136	0.382–3.384	0.818			
Diabetes	3.663	1.478–9.076	0.005	3.110	1.204–8.033	0.019
Hyperlipidemia	2.322	0.311–17.319	0.411			
Current smoker	0.783	0.263–2.328	0.659			
Hypertension	1.110	0.406–3.036	0.839			
Previous MI	0.798	0.322–1.978	0.626			
Previous PCI	1.627	0.674–3.928	0.280			
Previous CABG	3.035	1.256–7.336	0.014			
Previous stroke	3.241	0.952–11.035	0.060	5.490	1.494–20.177	0.010
Peripheral vascular disease	2.129	0.882–5.139	0.093			
Procedural success	0.282	0.116–0.683	0.005	0.349	0.137–0.886	0.027
eGFR (mL/min)	0.976	0.959–0.992	0.005	0.978	0.961–0.996	0.015
Chronic kidney disease	2.829	1.141–7.014	0.025			
LV ejection fraction (%)	0.975	0.928–1.024	0.308			
BMI	1.020	0.941–1.106	0.628			
Prior heart failure hospitalization	2.721	1.127–6.569	0.026			

HFH, heart failure hospitalization; MI, myocardial infarction; CABG, coronary artery bypass grafting; eGFR, estimated glomerular filtration rate; LV, left ventricular; BMI, body mass index; PCI, percutaneous coronary intervention; CTO, chronic total occlusion.

## 4. Discussion

In this analysis, from a prospective multicenter registry of patients with reduced LV function, we showed that: (1) CTO PCI is a procedure with a high success rate (82.5%), which can be performed with an acceptable number of complications, even in patients with higher baseline risks because of reduced LV ejection fraction(s); (2) at a median follow-up of 23 months, patients in the successful CTO PCI group had a lower risk of HFH compared to patients in the unsuccessful CTO PCI group, although no significance was observed in terms of WRF and AF; (3) in the multivariate analysis, successful CTO PCI was an independent predictor of reduced risk of HFH together with a higher eGFR, whereas prior stroke and diabetes were independent predictors of a higher risk of HFH.

Severe LVSD due to ischemic heart disease is associated with high morbidity, more frequent hospitalizations for heart failure, and deterioration of renal function, which have significant consequences on the prognosis and quality of life for patients. Previous registries have shown that a significant proportion of patients with ischemic LVSD have at least one CTO, and these patients have a higher frequency of prior MI, a higher prevalence of diabetes, and a higher percentage of triple vessel disease [2]. Collectively, these data suggest that patients with LVSD and at least one CTO have a very high risk of morbidity and mortality. In our study, at a median follow-up duration of 23 months, we observed overall and cardiovascular mortality rates consistent with previously published data, whilst 16% of patients were hospitalized for heart failure and 17.7% of patients experienced significant WRF, indicating the high-risk profile of these patients (the 2-year rates of HFH and WRF in the Enalapril group of the PARADIGM-HF study were 15.6% and 2.6%, respectively) [15].

Regarding the comparison between the successful and unsuccessful CTO PCI groups, patients in the unsuccessful CTO PCI group had more adverse baseline characteristics, both clinical and angiographic, compared to patients in the successful CTO PCI group. This difference in angiographic characteristics was expected since the J-CTO score, with its variables, is a recognized and widely used predictor of successful antegrade crossing within a short time and, indirectly, of CTO PCI success or failure [14]. The unsuccessful CTO PCI group also had a higher prevalence of CKD alongside previous HFH and CRT implantation. The higher CKD rate is not unexpected because it is generally associated with older and more challenging CTOs and a higher burden of calcifications within the occlusion. The higher prevalence of these factors in the unsuccessful PCI group suggests that these patients were possibly more complex and potentially characterized by a more advanced stage of disease than the patients in the successful PCI group. This could represent a possible confounding factor, although none of these variables were identified as independent predictors of the outcome during the multivariate analysis.

In this study, no significant differences were documented between the successful and unsuccessful CTO PCI groups in terms of rates of mortality, MI, target vessel failure, and stroke during the follow-up. For cardiovascular death, a trend toward a better outcome in the successful PCI group was clearly observed but it did not reach statistical significance. Other previously published larger registries [12, 18] have documented a reduction in mortality after successful CTO PCI in patients with LVSD, yet these data have never been explored in a specific randomized clinical trial.

Regarding clinical events related to heart failure, successful CTO PCI was associated with a reduced risk of HFH compared to unsuccessful CTO PCI, although there was no significant difference in terms of WRF and AF. This observation is both interesting and intriguing. However, since it was only formed from a registry with a small number of patients, currently, it should be considered only as hypothesis generating. Other registries that have studied CTO PCI in patients with LVSD have documented a prognostic benefit in terms of overall or cardiovascular mortalities but without an effect on MIs or major adverse cardiovascular events (MACE) [12, 18]. Therefore, it is possible that, at least in part, the prognostic benefit results from the improved heart failure clinical status of the patients. Indeed, during the follow-up, we observed improvements in NYHA and CCS classes and a slight improvement in ejection fraction. These improvements were consistent in both patients with successful CTO PCI and those with unsuccessful CTO PCI. Moreover, they could be explained by the positive effect of optimal medical therapy for heart failure, the adherence to which was optimal either at the time of recruitment or during the follow-up assessment. The number of patients in our study was not sufficient to conduct a reliable statistical analysis on further echocardiographic characteristics (diastolic function, filling pressures, mitral and tricuspid regurgitation, and right ventricular function) but it is known that ischemia is an important cause of diastolic dysfunction. This could be the underlying pathophysiological mechanism involved in the reduction of HFH. Hypothetically, these lower rates of HFH that were observed in the patients with LVSD could result from even a slight reduction in the extent of ischemic territory since this could lead to a limited improvement in systolic function. Moreover, it could also lead to an improvement in diastolic function, a reduction in filling pressures, and, consequently, in mitral regurgitation and pulmonary pressures, alongside an improvement in right ventricular function. However, this hypothesis is purely speculative and requires confirmation in dedicated studies using a larger cohort.

In the multivariate analysis of clinical events, successful CTO PCI and higher eGFR remained favorably associated with a lower risk of HFH during the follow-up. Conversely, a previous stroke and diabetes were associated with an increased risk of HFH. No other factors were independently associated with a reduction or increase in the risk of HFH during the follow-up period.

## 5. Conclusions

CTO-PCI is a procedure with a high success rate and acceptable complication rate in patients with LVSD and an ejection fraction ≤40%. In this cohort of patients, successful CTO PCI was associated with a reduced risk of HFH during the follow-up. Future studies with a larger patient cohort and longer follow-ups are needed to confirm these findings and further evaluate the benefits of CTO PCI in patients with LVSD.

## 6. Limitations

The results from this multicenter registry are observational and hypothesis-generating. We used patients with unsuccessful CTO PCIs as a control group to understand the effect of CTO PCI. Although these patients have very similar characteristics compared to patients with successful CTO PCIs, other confounding variables may still negatively affect the comparison (e.g., radiation exposure, contrast agent volume, and post-procedural hospitalization). The patients in the unsuccessful PCI group were possibly more complex than the patients in the successful PCI group (higher prevalence of prior HFH, CKD, and CRT implantation). Even though these were not identified as independent predictors of the outcome, they could still represent potential confounding factors.

Furthermore, the high success rate of CTO PCIs significantly reduced the number of patients in the unsuccessful CTO PCI group, thereby reducing the size of the control group.

Finally, the percentage of usage of AWE in comparison to ADR, RWE, and RDR appears higher than in contemporary practice. One might speculate that a potential reason is a mid-complexity range of the CTO lesions (average J-CTO score of 2.11) combined with the operator preferences being to avoid retrograde approaches to reduce the risk of donor vessel injury/ischemia in the setting of important LVSDs. Therefore, more prolonged antegrade attempts are possibly performed.

## Data Availability

The authors do not wish to share the complete dataset because of ongoing research on the same dataset.

## Supplementary Material

Supplementary material associated with this article can be found, in the online version, at https://doi.org/10.31083/j.rcm2412345.
