# Supplementary material for: Heart Failure-Related Outcomes in Patients with Left Ventricular Dysfunction Undergoing Percutaneous Chronic Total Occlusion Revascularization
Source: Rev Cardiovasc Med. 2023 Dec 12;24(12):345. doi: 10.31083/j.rcm2412345 (PMC11262444; doi:10.31083/j.rcm2412345)

Supplementary Table 1. .Clinical and echocardiographic characteristics during follow-up.

|  | Entire cohort | Successful PCI (n=108) | Non successful PCI (n=23) | Statistical difference (p value) |
| --- | --- | --- | --- | --- |
| NYHA class  1  2  3  4 | 52 (50)  39 (37.5)  12 (11.5)  1 (1.0) | 45 (51.7)  31 (35.6)  10 (11.5)  1 (1.1) | 7 (41.2)  8 (47.1)  2 (11.8)  0 (0.0) | 0.404 |
| CCS class  No angina  1  2  3  4 | 95 (81.2)  19 (16.2)  3 (2.6)  0 (0.0)  0 (0.0) | 77 (77.8)  19 (19.2)  3 (3.0)  0 (0.0)  0 (0.0) | 18 (100)  0 (0.0)  0 (0.0)  0 (0.0)  0 (0.0) | **< 0.001** |
| Systolic blood pressure, mmHg | 123.7 ± 19.6 | 124.5 ± 20.3 | 119.6 ± 15.3 | 0.283 |
| Heart rate, bpm | 68.5 ± 12.6 | 68.6 ± 13.1 | 67.5 ± 8.8 | 0.672 |
| Sinus rhythm | 104 (88.1) | 88 (88.0) | 16 (88.9) | 0.915 |
| Creatinine, mg/dl | 1.39 ± 0.87 | 1.41 ± 0.93 | 1.31 ± 0.41 | 0.527 |
| eGFR, ml/min/1,73m2 | 58.0 ± 23.0 | 58.8 ± 23.4 | 54.1 ± 21.1 | 0.461 |
| Left Ventricular Ejection fraction, % | 39.4 ± 11.0 | 40.1 ± 11.3 | 35.4 ± 8.6 | 0.065 |
| Right ventricular dysfunction | 16 (17.6) | 12 (15.8) | 4 (26.7) | 0.312 |
| Mitral regurgitation grade >2 | 3 (3.0) | 2 (2.4) | 1 (6.7) | 0.367 |
| Tricuspide regurgitation grade >2 | 3 (3.1) | 3 (3.7) | 0 (0.0) | 0.452 |
| Treatment  ACEi/ARB/LCZ696  Beta-blocker  ARA  Diuretics  Digoxin | 101 (76.5)  109 (90.1)  58 (47.9)  55 (45.5)  2 (1.7) | 84 (77.1)  94 (90.4)  46 (44.2)  45 (43.3)  2 (1.9) | 17 (73.9)  15 (88.2)  12 (70.6)  10 (58.8)  0 (0.0) | 0.746  0.783  **0.044**  0.232  0.564 |
| Implantation of ICD | 18 (13.8) | 14 (13.1) | 4 (17.4) | 0.587 |
| Implantation of CRT | 14 (10.8) | 11 (10.3) | 2 (13.0) | 0.698 |
| NYHA: New York Heart Association; CCS: Canadian Cardiovascular Society; eGFR: estimated glomerular filtration rate; ACEi: angiotensin-converting enzyme inhibitor; ARB: angiotensin receptor blocker; ARA: aldosterone receptor antagonist; ICD: implantable cardioverter-defibrillator; CRT: cardiac resynchronization therapy. | | | | |

Supplementary Fig. 1. Events during follow-up


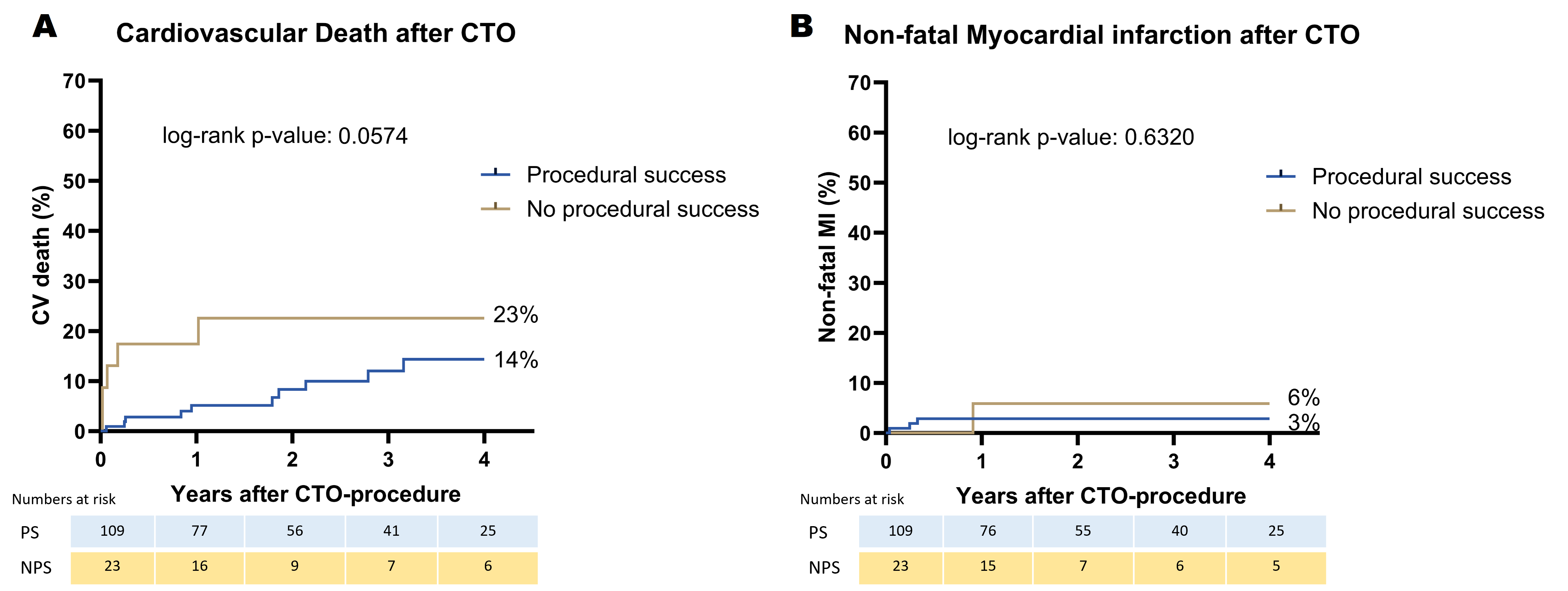

Supplement: Supplementary file 1 [file 2153-8174-24-12-345-s1.docx]
